# Challenges in diagnostics and treatment of infant-type hemispheric gliomas

**DOI:** 10.1093/noajnl/vdaf124

**Published:** 2025-07-30

**Authors:** Ludmila Papusha, Margarita Zaytseva, Maria Senchenko, Agnesa Panferova, Agunda Sanakoeva, Anton Artemov, Alexandra Korostyshevskaya, Alexandra Tarakanova, Djamilya Murzaeva, Igor Kasich, Artur Merishavyan, Andrey Flegontov, Ekaterina Salnikova, Artem Zaychikov, Inna Proleskovskaya, Yulia Dinikina, Zhanna Kumykova, Anastasia Protsvetkina, Natalia Usman, Alexander Karachunskiy, Eugene I Hwang, Roger J Packer, Galina Novichkova, Alexander Druy

**Affiliations:** D. Rogachev National Medical Research Center of Pediatric Hematology, Oncology and Immunology, Moscow, Russia; D. Rogachev National Medical Research Center of Pediatric Hematology, Oncology and Immunology, Moscow, Russia; D. Rogachev National Medical Research Center of Pediatric Hematology, Oncology and Immunology, Moscow, Russia; D. Rogachev National Medical Research Center of Pediatric Hematology, Oncology and Immunology, Moscow, Russia; D. Rogachev National Medical Research Center of Pediatric Hematology, Oncology and Immunology, Moscow, Russia; D. Rogachev National Medical Research Center of Pediatric Hematology, Oncology and Immunology, Moscow, Russia; International Tomography Center of the Siberian Branch of the Russian Academy of Sciences, Novosibirsk, Russia; D. Rogachev National Medical Research Center of Pediatric Hematology, Oncology and Immunology, Moscow, Russia; Pavlov First Saint Petersburg State Medical University, Saint Petersburg, Russia; D. Rogachev National Medical Research Center of Pediatric Hematology, Oncology and Immunology, Moscow, Russia; D. Rogachev National Medical Research Center of Pediatric Hematology, Oncology and Immunology, Moscow, Russia; D. Rogachev National Medical Research Center of Pediatric Hematology, Oncology and Immunology, Moscow, Russia; D. Rogachev National Medical Research Center of Pediatric Hematology, Oncology and Immunology, Moscow, Russia; Regional Children’s Hospital, Yekaterinburg, Russia; Republican Scientific and Practical Center for Children’s Oncology, Hematology and Immunology, Minsk, Belarus; Almazov National Medical Research Centre, Saint Petersburg, Russia; Republican Children’s Clinical Multidisciplinary Center, Nalchik, Russia; D. Rogachev National Medical Research Center of Pediatric Hematology, Oncology and Immunology, Moscow, Russia; D. Rogachev National Medical Research Center of Pediatric Hematology, Oncology and Immunology, Moscow, Russia; D. Rogachev National Medical Research Center of Pediatric Hematology, Oncology and Immunology, Moscow, Russia; Brain Tumor Institute, Children’s National Hospital, Washington, DC, USA; Division of Oncology, Children’s National Hospital, Washington, DC, USA; Center for Neuroscience and Behavioral Medicine, Children’s National Hospital, Washington, DC, USA; Brain Tumor Institute, Children’s National Hospital, Washington, DC, USA; D. Rogachev National Medical Research Center of Pediatric Hematology, Oncology and Immunology, Moscow, Russia; Research Institute of Medical Cell Technologies, Yekaterinburg, Russia; D. Rogachev National Medical Research Center of Pediatric Hematology, Oncology and Immunology, Moscow, Russia

**Keywords:** entrectinib, infant-type hemispheric gliomas, lorlatinib, targeted therapy, tyrosine-kinase fusions

## Abstract

**Background:**

Infant-type hemispheric gliomas (IHG) represent a novel entity, first codified in the WHO CNS 5 classification. Due to their rarity, as well as their neuroimaging and histopathologic heterogeneity, definitive diagnosis can be challenging. In the majority of cases, the tumors are large, and difficult to fully resect. The efficacy of standard cytotoxic chemotherapy remains unclear. IHGs frequently contain receptor tyrosine-kinase (RTK) gene fusions, denoting a potential vulnerability to targeted therapy by small-molecule RTK inhibitors.

**Methods:**

We report 15 patients with IHG receiving treatment during a 5-year period. Integrated diagnosis was achieved combining histopathology, DNA methylation profiling and RNA sequencing. Ten out of 15 patients received chemotherapy. Targeted therapy with entrectinib or lorlatinib was prescribed in 5 patients after progression and in 1 as first-line treatment.

**Results:**

The median follow-up was 1.5 years (range, 0.1-5.1 years). Six patients were asymptomatic despite large volumes and diagnosed during routine ultrasound screening. Neuroimaging revealed 2 general radiographic presentation, either cystic-solid or purely solid masses. These radiologic subtypes were not associated with differences in histology or clinical behavior, but demonstrated differential gene expression profiles. Standard cytotoxic chemotherapy was administered in 10 patients, in 6 of them disease progression was observed (all with residual tumor). RTK gene fusions were revealed in all cases. Six patients were treated with targeted therapies. All patients had an initial tumor response; following which 2 had disease progression. One-year event-free survival for the entire cohort was 47% (CI 27%-80%), 2-year overall survival was 61% (CI 39%-95%).

**Conclusions:**

IHGs are comprised of 2 radiologically and molecularly distinct groups. Huge cystic tumors are frequently associated with life-threating complications. The limited efficacy of the standard cytotoxic chemotherapy and presence of kinase fusion in nearly all cases render patients with IHG candidates for targeted therapies.

**Key Points**

- IHGs harbor tyrosine-kinase fusions indicating potential sensitivity to targeted therapy
- Many of patients were asymptomatic and tumors were incidentally detected on the ultrasound screening
- IHGs are comprised of 2 radiologically distinct groups with different gene expression profile

**Importance of the Study**

Infant-type hemispheric gliomas (IHG) present challenges for diagnostics and cure. Highly vascularized large tumors often cannot be safely removed. The diagnostics of IHG requires molecular characterization of the tumor, as many cases microscopically resemble desmoplastic infantile ganglioglioma/astrocytoma, astroblastoma or ependymoma.

Despite the extensiveness of these tumors at diagnosis, patients were often asymptomatic. The lesions are often found incidentally at neurosonography screening. MRI appearance of IHGs revealed 2 distinct groups—cystic-solid and purely solid masses. Patients with abundant cystic component in the tumor were at risk of perioperative life-threating bleeding. Tumors belonged to these radiographic subtypes had different gene expression profiles.

In all cases actionable tyrosine-kinase fusion was detected and 6 children received treatment with entrectinib or lorlatinib. All recipients have responded to therapy, while subsequent tumor regrowth was seen in 2.

Infant-type hemispheric gliomas (IHG) are rare high-grade astrocytic tumors characterized by giant size and abundant vascularity, often with regions of cystic transformation. They are typically first noted in infancy or in utero.^1–3^ Although standard clinical options for IHG include surgery and polychemotherapy, these tumors have limited sensitivity to chemotherapy, and their size and vascularity pose significant surgical risks.^4,5^ Nevertheless, survival rates in IHG are higher for other pediatric high-grade gliomas.^1^

Morphological diagnosis in IHG is complicated due to their rarity and lack of specific histological hallmarks. In the majority of cases, immunohistochemistry reveals astrocytic differentiation. However, the appearance of perivascular cell clusters in IHG can be diagnostically misleading, as similar rosette-like patterns are found in other malignant brain tumors (astroblastoma, ependymoma). The varying grade of malignancy may complicate differentiation from desmoplastic infantile gangliogliomas/astrocytomas (DIG/DIA) which are similar to IHG in morphology and age at diagnosis.^3^

Biologically, IHG are distinguished by specific driver rearrangements that preserve the catalytic domain-encoding sequence of a receptor tyrosine-kinase (RTK) gene in-frame with a 5′ fusion partner. The driver rearrangements in IHG may involve different RTK genes; most typically *ALK*, more rarely *NTRK* family or *ROS1*, and in isolated cases *MET*.^6^ The rearrangements in IHG invariably involve genes of membrane tyrosine kinases but not cytoplasmic serine-threonine kinases, a distinction which aids in differentiation from DIG/DIA. Identification of a pathognomonic chimeric transcript provides a highly specific diagnostic marker and is also a target for molecularly tailored therapy. Recent studies demonstrate the efficacy of targeted therapy for high-grade gliomas in different age groups.^6^ In particular, the use of RTK inhibitors had limited toxicity and was efficacious in patients with *NTRK*-rearranged IHG and *ROS1*-positive gliomas treated on the STARTRK-NG trial or as reported in case reports.^7–12^ ALK inhibitors in *ALK*-rearranged IHG were also efficacious.^13–16^

This study summarizes the radiological, morphological and molecular-genetic findings in IHG, as well as clinical outcomes after the use of chemotherapy and molecularly targeted therapeutics.

## Patients and Methods

The study was approved by the independent Ethics Committee and ratified by the Academic Council at the Dmitry Rogachev National Medical Research Center of Pediatric Hematology, Oncology and Immunology. The enrollment was based on morphologically verified high-grade astrocytic glioma of hemispheric localization in patients less than 2 years of age. Informed voluntary consent for participation in the study was provided for all patients by their parents or legal representatives.

The study enrolled 15 consecutive patients seen at our institution, including 2 patients (22 months, 24 months old) initially diagnosed with anaplastic ependymoma and astroblastoma, based on histological findings. The latter 2 tumors were found to harbor RTK gene fusions and did not have evidence of *YAP1*, *ZFTA* or *BEND2* rearrangements; these patients were retrospectively included into the study. The integrated diagnosis of infant-type hemispheric glioma was made on the basis of morphological and molecular-genetic characterization of the tumor in accordance with the 2021 WHO Classification of CNS Tumors.

Molecular-genetic studies included RNA sequencing with RNA Exome Panel (Illumina) at 2 × 100 paired-end reads in a NextSeq 550Dx instrument (Illumina), followed by data analysis in Dragen RNA Pipeline v.3.6.3 and Arriba v.2.4.0 applications.^17^ The DNA methylation profile assignment to reference tumor methylation classes and the assessment of unbalanced genomic aberrations (copy number variations, CNV) were performed using Infinium MethylationEPIC v1.0/2.0 (Illumina) and the Molecular Neuropathology Brain Tumor Classifier version 12.8 (DKFZ). Gene expression heterogeneity was assessed with nCounter^®^ PanCancer Pathways Panel (NanoString); the data were analyzed using the ROSALIND platform.

Ten of 15 children initially received chemotherapy (CT) in accordance with BabyPOG (6/10 cases) and HIT-SKK protocols (4/10 cases). Targeted therapy with entrectinib was administered to 4 of 6 patients at the time of disease progression. Lorlatinib was used as first-line medication in 1 and in 2 patients at the time of progression (either after chemotherapy or on entrectinib). The response to the treatment was assessed by RAPNO criteria; adverse events were classified using CTCAE v5.0.^18,19^

## Results

### Clinical signs

**Figure 1.**
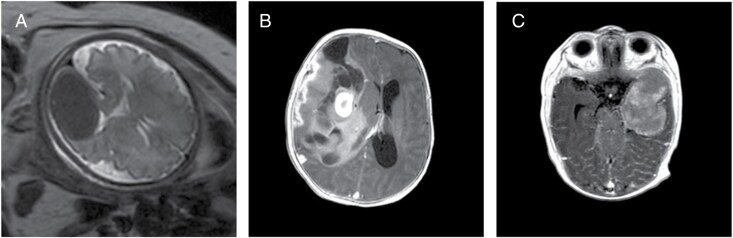
(A) T2-weighted fetal MRI showing hemispheric solid mass in the occipital lobe; (B and С) T1-weighted MRI with contrast enhancement of cystic-solid (Rӧ-subtype 1, B) and solid (Rӧ-subtype 2, C) IHG.

Age at diagnosis ranged from 2 days to 22 months, median 3 months, with a predominance of females (female to male ratio of 2:1). 7 of 15 children were asymptomatic, as the tumor was detected during routine screening neurosonography (*n* = 6) or as an incidental finding (*n* = 1). In 1 case, a fetal brain tumor was detected during a routine ultrasound examination at 32 weeks of gestation and was subsequently confirmed by prenatal MRI (Fig. 1A). In 7 cases, the tumors were symptomatic causing signs of increased intracranial pressure in 5, seizures in 1, and hemiparesis in 1.

### Radiographic Features

Tumors in general had 2 different radiographic appearances. The first, radiological (Rӧ) subtype 1, displayed a cystic-solid tumor without intensive contrast enhancement along the cyst walls, but with significant enhancement of the solid component (Fig. 1B). In 3 patients, tumors of this radiological subtype were giant (V = 240–650 cm^3^), occupied an entire hemisphere, and had multiple intratumoral hemorrhages. Lesions of the second subtype (Rӧ-subtype 2) had a purely solid structure with distinct contours and homogeneous with medium-intensity contrast enhancement (Fig. 1C).

All tumors were large in volume (ranging from 9.4cm^3^ to 650cm^3^, median 107cm^3^) with minimal peritumoral edema; none were metastatic. Intratumoral hemorrhage was common with findings consistent with previous or active hemorrhage. Restricted diffusion was evident in all cases.

### Surgical Aspects

Resection was performed in all cases. In order to minimize surgical complications, pre-surgical management attempted in 4 cases, all in children less than 4 months of diagnosis. Total or near total resections (residual tumor volume less than 1.5 cm^3^) were performed in 8 patients. The remainder had subtotal resections. A total of 27 craniotomies were performed on the cohort, with 8 patients experiencing 2 or more surgical interventions. In 2 cases, the surgery was complicated by hemorrhage into the residual tumor, causing cerebral edema and death.

### Morphology

**Figure 2.**
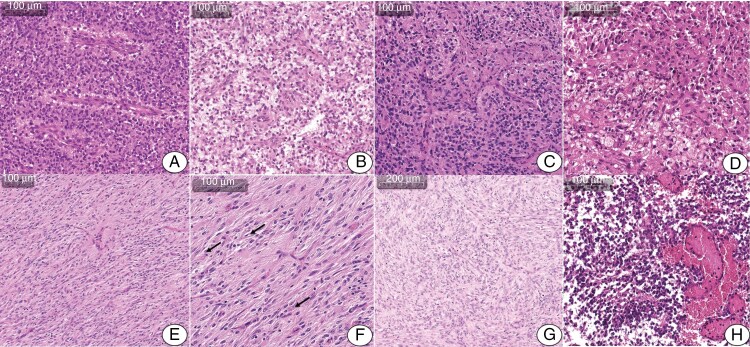
Histological synopsis for IHG. (A and B.) Signs of pronounced gemistocytic differentiation; perivascularly located cells form pseudorosette structures of varying order giving a resemblance to ependymoma (#7, A) or astroblastoma (#3, B). (С) Vascular endothelial proliferation (#8). (D) Focal accumulations of xanthomatous cells, some containing hemosiderin (#2). (E and F) Hypercellular neoplastic tissue composed of spindle-shaped, slightly polymorphic cells (#9); arrows indicate solitary cells with signs of gemistocytic differentiation. (G) Hypercellular, spindle-cell, neoplastic tissue without signs of gemistocytic differentiation (#5). (H) Full-blooded vessels without signs of endothelial proliferation amid rounded hyperchromic tumor cells, focal palisade necrosis (#6).

Histological examination revealed evidence of well-demarcated tumor growth in all cases, with high or moderate density of cellular elements. Eleven showed varying degrees of gemistocytic differentiation (Fig. 2A). Widespread gemistocytic differentiation was observed in 9 cases (Fig. 2A to D). In cases #8 and #9, isolated large gemistocytes amid the spindle-cell component suggested a morphological similarity to DIG/DIA (Fig. 2E and F). Single cases presented with focal stromal desmoplasia or purely spindle-cell neoplastic tissue without signs of gemistocytic differentiation (#5, Fig. 2G).

The radiological distinctions of the tumors had no correlates at the microscopic level. Three giant cystic-solid tumors of Rӧ-subtype 1 were histologically distinct; 2 of them revealed gemistocytic differentiation typical for the studied cohort, and 1 tumor revealed spindle-cell morphology.

All tumors were noted to have high vascularity, predominantly thin-walled vessels with focal endothelial proliferation (Fig. 2C) observed in cases #7, #8, #11 and #12. Varying degrees of perivascular clustering was observed in 12 cases, resembling than seen in astroblastomas or ependymomas (Fig. 2A and B). Focal palisading necrosis was observed in 2 cases (Fig. 2H). Mitotic activity ranged from 2 to greater than 10 mitotic figures per 10 high-power fields × 400. About half of the cases presented with focal hemorrhage, with or without hemosiderophages (Fig. 2D).

Immunohistochemistry revealed diffuse S100 positivity in all cases (15/15). The GFAP expression was either diffuse (*n* = 11) or focal (*n* = 4). Olig2 was negative in 7 cases; in cases #4, #11 and #13 approximately one-half of tumor cells were Olig2-positive. Case #7 had dot-like positivity for pan-cytokeratin expressed by solitary cells. Dot-like expression of EMA was observed in 3 cases, in 1 focal membranous expression was observed. All tumors were immunohistochemically negative for CD34. Synaptophysin and Chromogranin A expressions were noted in tumors with neuronal differentiation. The anti-NF reactions stained single residual axons mainly at the tumor periphery.

Four cases demonstrated histological differences from the rest of the cohort. In case #6 microscopic examination revealed a hypercellular neoplastic tissue composed of rounded, slightly polymorphic cells with a high nuclear-cytoplasmic ratio and hyperchromatic nuclei (Fig. 2H). In case #9 the tumor had featured characteristics of low-grade gliomas with predominance of spindle-cell elements associated with solitary, discretely located gemistocytic cells. In this case there was the lack of stromal desmoplasia, necrosis or vascular endothelial proliferation and a mitotic activity level of less than 2 mitotic figures per 10 high-power fields × 400 (hence the initial diagnosis of DIA). In cases #10 the tumor revealed minor foci of high mitotic activity with pronounced endothelial proliferation amid a dominant low-grade component. In this case the diagnosis was retrospectively changed from DIA to IHG. In case #15 the low-grade glioma component predominated with interspersed neuronal / ganglionic cells and foci of high mitotic activity. The described cases of IHG with atypical morphology belonged to different radiological subtypes.

### Molecular-genetic characterization

A pathogenic rearrangement involving an RTK gene was revealed in all tumors of the cohort. The affected RTK genes included *ROS1* (*n* = 5), *ALK* (*n* = 3), *NTRK1* (*n* = 3), *NTRK3* (*n* = 3) and *EGFR* (*n* = 1). The rearrangements produced fusions with miscellaneous 5′ partners; in particular, *ROS1* catalytic domain-encoding sequence was fused to *ZCCHC8* (*n* = 2), *ZBTB48*, *KLC1* or *SRSF5*. Other 5′ fusion partners included *TPR* (*n* = 2, both in rearrangement with *NTRK1*), *ETV6* (*n* = 2) and *VIM*, both in rearrangement with *NTRK3*, *QKI*, *BTNL8* and *PPP1CB* (fused to *ALK*). In all cases, the complete catalytic domain-encoding sequence was preserved in-frame with the fusion partner. In case #12, *NTRK1* rearrangement with an unknown partner was deduced from a 5′/3′ expression imbalance for *NTRK1*. Case #6, markedly distinct histologically from the rest of the cohort, harbored a *CLIP2*::*EGFR* rearrangement atypical for IHG.

In all but 2 of the cases, epigenetic profiles identified with the Infant-Type Hemispheric Glioma methylation class with a matching score of > 0.90. In cases #1 and #11, the matching scores of, respectively, 0.63 and 0.43 were diagnostically non-significant, however both cases were diagnosed as IHG based on integrated histomolecular findings.

Morphological, molecular-genetic and radiographic findings for the cohort are summarized in Table 1.

**Table 1.** Morphological, molecular-genetic and radiographic characterization of the tumors

Pt#	Age, months	Histological diagnosis	Fusion transcript	Methylation class BTCv12.8	BTC v12.8 score	CNV	Rö-subtype
1	1	AA grade 3	*ZCCHC8::ROS1*	MC infant-type hemispheric glioma	0.63	−3, −6q (focal), −10, −11, −22, +8q	2
2	4	glioblastoma/PXA	*ETV6::NTRK3*	MC infant-type hemispheric glioma	0.95	+5, +8, +12p, +15q, +17	1
3	0.1	IHG/ astroblastoma	*ZCCHC8::ROS1*	MC infant-type hemispheric glioma	0.99	−6q (focal), +8q	1
4	22	Astroblastoma	*QKI::ALK*	MC infant-type hemispheric glioma	0.91	−16, −19, −22	1
5	1	IHG	*TPR::NTRK1*	MC infant-type hemispheric glioma	0.99	Flat profile	1
6	6	HGG	*CLIP2::EGFR*	MC infant-type hemispheric glioma	0.96	+7p/+7q (focal paracentric), homozygous deletion *CDKN2A/B*	1
7	2	AE grade 3	*BTNL8::ALK*	MC infant-type hemispheric glioma	0.97	Flat profile	1
8	4	IHG	*ZBTB48::ROS1*	MC infant-type hemispheric glioma	0.99	Flat profile	1
9	2	DIA/IHG	*KLC1::ROS1*	MC infant-type hemispheric glioma	0.99	−19	2
10	9	DIA/IHG	*TPR::NTRK1*	MC infant-type hemispheric glioma	0.93	−4, −6, −15	1
11	18	IHG	*ETV6::NTRK3*	MC infant-type hemispheric glioma	0.43	+1q, +7, +8, +9, +19, +20	2
12	0.5	IHG	*NTRK1* with unknown partner gene	MC infant-type hemispheric glioma	0.99	−7	2
13	12	IHG	*SRSF1::ROS1*	MC infant-type hemispheric glioma	0.96	−6q (focal), +12, +18	2
14	3	IHG	*VIM::NTRK3*	MC infant-type hemispheric glioma	0.99	−7p (focal), 19p/−19q (focal paracentric)	1
15	2	DIA/IHG	*PPP1CB::ALK*	MC infant-type hemispheric glioma	0.95	High level 2p23.2 amplification	2

AA, anaplastic astrocytoma; IHG, infant-type hemispheric glioma; HGG, high-grade glioma; AE, anaplastic ependymoma; DIG/DIA, desmoplastic infantile ganglioglioma/astrocytoma. PXA, pleomorphic xanthoastrocytoma; MC, methylation class; BTC, Brain Tumor Classifier version 12.8 (DKFZ); CNV, copy number variations; Rö-subtype, roentgenological subtype of the tumor (1: cystic-solid; 2: solid).

In 9/15 cases, the tumors had aneuploidy karyotypes with multiple numerical and segmental chromosomal aberrations. In cases #1, #3 and #13 the *ROS1*-rearranged tumors harbored focal deletions of 6q associated with the rearrangement, involving 6q22.1 locus containing *ROS1* gene. In cases #1 and #3 the *ZCCHC8::ROS1* fusions were accompanied by increased copy number of the 8q region. In case #6, the *CLIP2*::*EGFR* fusion was accompanied by a focally increased copy number of pericentromeric region in chromosome 7, involving the 7p11.2 locus containing *EGFR*. In case #15 high level amplification of the chromosomal locus 2p23.2 provided the basis for *PPP1CB::ALK* fusion formation. Flat cytogenetic profiles were encountered in 3 cases.

**Figure 3.**
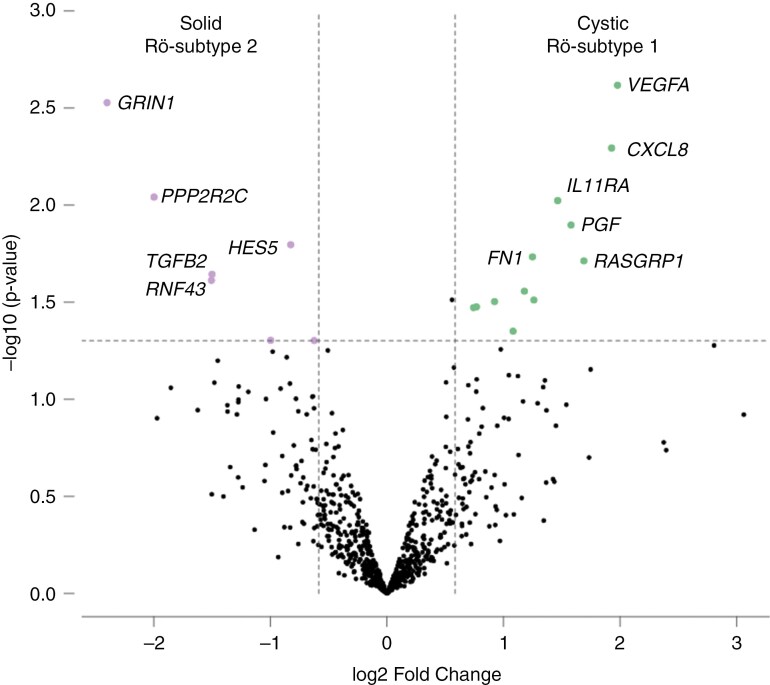
Volcano plot of differentially expressed genes in Rӧ-subtype 1 (cystic-solid) and Rӧ-subtype 2 (solid) IHGs

The fusion transcript identities, as well as the numerical and segmental chromosomal aberration profiles, did not correlate with the roentgenological (Rö) subtype of the tumor. In search for molecular correlates of the phenotypic diversity in IHG, genes implicated in oncogenic signaling pathways were assessed for differential expression using NanoString. Despite the lack of significant matches to Rö-subtype produced by unsupervised hierarchical clustering of the data, the differential gene expression analysis (Fold change > 1.5, < -1.5, False discovery rate < 0.05) revealed elevated mRNA levels for several oncogenes (*FOS*, *NGF*, *PGF*, *RASGRP1*, *TCF7L1*), tumor suppressors (*GAS1*, *MAP3K20*) and paracrine regulators/cell-matrix interaction proteins (*VEGFA*, *IL3RA*, *IL11RA*, *CXCL8*, *FN1*) in cystic-solid tumors (Rö-subtype 1). In solid tumors (Rö-subtype 2), the analysis revealed elevated mRNA levels for *GRIN1*, *RNF43*, *HES5*, *TGFB2*, *PPP2R2C*, *SMC1B* and *CCND2* compared to Rö-subtype 1 (Fig. 3). Affiliation of the differentially expressed genes to particular signaling pathways and biological processes indicated the enhancement of VEGF-dependent angiogenesis and interleukin signaling in Rö-subtype 1 compared with Rö-subtype 2. Furthermore, activation of *TCF7L1* encoding a transcription factor that mediates WNT signaling and antagonizes the TGFβ pathway in cystic-solid IHG (Rö-subtype 1) suggested a pathogenetic difference from solid IHG (Rö-subtype 2) that harbor increased expression of a WNT antagonist *RNF43* and also of *TGF2B* encoding a secreted TGFβ ligand. Of note, the differentially expressed *VEGFA*, *NGF*, *CXCL8*, *HES5* and *GRIN1* affiliate to the sudden infant death syndrome susceptibility pathway.

### Therapy and clinical outcomes

The median follow-up time was 1.5 years (range 0.1-5.1 years). Three of the cases enrolled in the study have been previously reported with a more limited follow-up period.^20,21^

**Figure 4.**
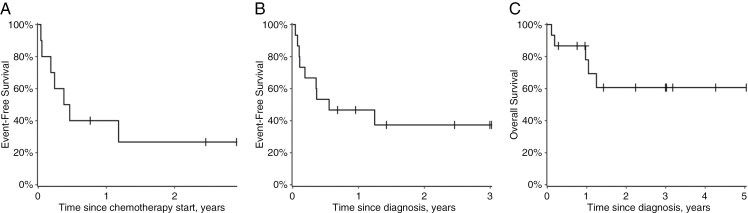
(A) Event-free survival in patients with IHG on cytotoxic chemotherapy. (B and C) Event-free (B) and overall (C) survival rates for the studied cohort of patients with IHG.

In 10/15 cases, the patients received standard cytotoxic chemotherapy in accordance with BabyPOG (*n* = 6) or HIT-SKK (*n* = 4) protocols. Two patients were spared adjuvant therapy after radical resection because their histological diagnosis was initially formulated as DIA. At the time of manuscript acceptance, both patients have maintained disease remission for 3 and 1.4 years. One patient with a *SRSF1*::*ROS1* rearrangement continues to receive targeted therapy after treatment with frontline lorlatinib. Disease progression was recorded in 6/10 chemotherapy recipients and occured solely in patients with residual tumor after operation. One patient died of septicemia 4 months after the end of chemotherapy, without signs of disease progression. One-year event-free survival (EFS) in patients with IHG on cytotoxic chemotherapy was 40% (CI 19%-85%) (Fig. 4A).

Of 6 patients who developed disease progression on the chemotherapy, 1 child died and the other 5 were begun on molecularly targeted therapy: entrectinib (*n* = 4) or lorlatinib (*n* = 1). The inhibition was targeted at fusion proteins *ZCCHC8::ROS1* (*n* = 2), *ZBTB48::ROS1* (*n* = 1), *ETV6::NTRK3* (*n* = 1) or *PPP1CB::ALK* (*n* = 1). Two recipients had a complete tumor response after 1.5 month and 6 month on treatment, respectively and 1 other patient had partial response. Two had stable disease. Responses were rapid and response was noted within 4 months of therapy in all. The median time on entrectinib was 20 months (range 8-48 months). The duration of continuing therapy with lorlatinib is 5 months. A previously described female patient with *ETV6::NTRK3* rearranged tumor^21^ completed 48 months of entrectinib therapy, with a sustained complete response. The patient developed osteopenia complicated by a compression fracture of the T8 vertebral body 2 years into therapy and required short-term breaks due to grade 3 neutropenia. Ultimately the child required a dose reduction. Neurologically, the same patient also developed epileptic encephalopathy and is believed to be secondary to surgery-associated organic brain damage. The patient remains in remission for 6 months after stopping treatment.

A previously described female patient with a *ZCCHC8::ROS1* rearranged tumor who had a complete response recorded 12 months into RTK inhibition regimen^20^ is currently maintaining the remission 12 months after completing 18 months of entrectinib therapy. In this child therapy was complicated by a decrease in left ventricular ejection fraction to a minimum of 36% by Teichholz, which required a 2 week break in the regimen, followed by full-dose resumption. In another patient with similar *ZCCHC8::ROS1* rearrangement, also included in the same report,^20^ entrectinib *resulted in* reduction in tumor volume. There was significant improvement of the neurological status recorded 4 weeks into therapy, which allowed continued treatment on an outpatient basis. However, 8 months into therapy, rapid progression of the disease occurred and repeat surgical resection was complicated by a massive fatal bleeding.

Similarly, in a patient with *ZBTB48::ROS1* rearranged IHG who had failed chemotherapy, entrectinib therapy resulted in a 35% reduction in tumor volume over the course of 2 months. However, 6 months into therapy, disease progression was evident. Lorlatinib, a more potent RTK inhibitor active against ROS1 tyrosine kinase, was commenced after a repeat partial resection of the tumor. Despite a partial response, with an 81.3% reduction in tumor volume, recurrent disease progression was evident 3 months into therapy. The use of lorlatinib was accompanied by significant weight gain in the patient, regarded as a grade 2 adverse effect.

Two patients with IHG harboring *QKI::ALK* and *CLIP2::EGFR* rearrangements are currently in an ongoing complete remission lasting, respectively, 27 and 22 months after radical resection of the tumor and completion of full treatment course of chemotherapy.

Overall, 1-year EFS for the cohort was 47% (CI 27%-80%), Fig. 4B. The 2-year overall survival was 61% (CI 39%-95%), Fig. 4C. At the time of writing, 10 children survived and 5 children died either of surgical complications from initial surgery (*n* = 2), progression on chemotherapy (*n* = 1), progression on targeted therapy (*n* = 1) or infectious complication during remission (*n* = 1). No differences in survival with regard to roentgenological subtype of IHG were noted.

**Figure 5.**
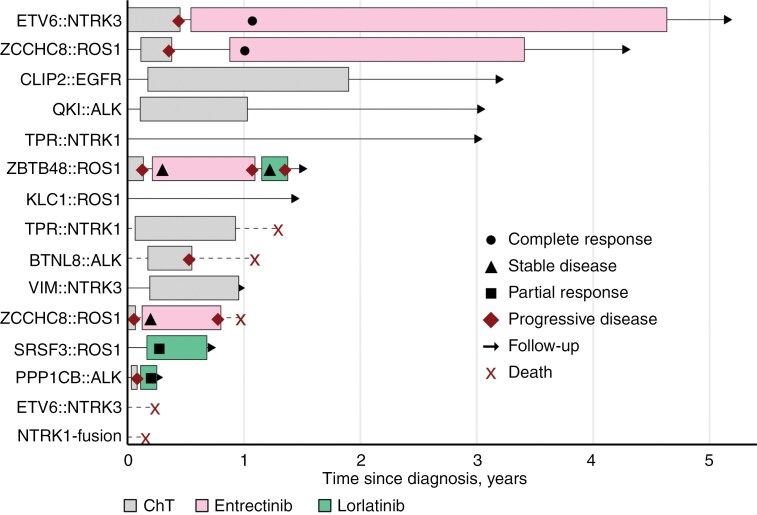
Swimmer plot of clinical course and therapeutic interventions for the studied cohort with IHG.

Chronological visualization of clinical course and the therapies is presented in Fig. 5.

## Discussion

Infant-type hemispheric glioma (IHG), a rare primary brain tumor, presents difficulties in both diagnosis and therapy. The entity was characterized molecularly in 2019, although isolated descriptions of glial tumors with similar MRI features (giant tumor size, cystic degeneration, hemorrhages) and histological assignment (ependymoma-like glioma) were published earlier.^6,22^ The current 2021 WHO Classification of CNS Tumors identifies IHG as independent entity.^23^

The disease typically manifests during the first year of life. Some patients clinically present with an increase in head circumference, although many cases are initially asymptomatic, despite the significant tumor volume, and are incidentally detected by routine screening neurosonography. In Russia, ultrasound brain examination is a mandatory procedure for all newborns at the age of 1 month. The primary aim of this screening is an early detection of the hydrocephalus and congenital brain malformations. The high prevalence of asymptomatic patients with IHGs in the studied cohort (40%, 6/15) underscores the utility of screening measures for particular pediatric cancers.

In certain cases, IHG can be detected in utero during the second trimester of gestation or later.^24,25^ In prenatal ultrasound scans, IHG are visualized as large homogeneous solid masses located hemispherically, ovoid in shape, resembling a hematoma or a thrombosed venous sinus. In case #12 of this study, the MRI revealed a fairly homogeneous solid structure in the fetal brain with apparent small foci of necrosis and hemorrhage and hypo- and hyperintense signals in T2- and T1-WI, respectively; similar findings were reported by Tsai et al. and Cornejo et al.^24,25^

Analysis of MRI features of IHG for the studied cohort distinguished 2 roentgenological subtypes: (1) cystic-solid with hemorrhages and intense contrasting of the solid component; and (2) solid with fairly clear smooth contours and homogeneous contrasting of medium-intensity. However, radiologic subtyping did not correlate with histological or clinical features.

Difficulties of morphological diagnosis in IHG are related to the rarity of the condition and its similarity with other tumors.^3^ The biggest challenge in differential diagnosis between IHG and DIG/DIA is posed by the prevalence of spindle-cell components in certain IHGs^3^; IHG are distinguished by gemistocytic differentiation, vessels with signs of endothelial proliferation, hemorrhages with focal accumulations of hemosiderophages, palisading necrosis, the absence of stromal desmoplasia and the increased mitotic activity.

The morphological similarity of IHG with other tumors necessitates molecular phenotyping, especially regarding RTK gene rearrangements, along with methylation profiling. The identity of the specific genes involved in the pathogenic rearrangement can impact molecular-based management.

Molecular-genetic correlates of the differential MRI semiotics in IHG were found at the transcriptomic level. The highly vascularized cystic tumors with often hemorrhages (Rö-subtype 1) showed transcriptomic signs of interaction with extracellular matrix and reactive microenvironments, involving the interleukin and chemokine signaling. The high expression of *VEGFA* in cystic IHG is consistent with extremely high rates of angiogenesis in these tumors which intraoperatively may appear as abundant gelatinous mass on numerous branched blood vessels. Differential expression of WNT and TGFβ pathway regulators was also noted, with a WNT agonist and TGFβ antagonist *TCF7L1* upregulated in cystic IHG, and a WNT antagonist *RNF43* and a TGFβ agonist *TGFB2* upregulated in solid IHG. The multiple genes differentially expressed in some subtypes of IHG, are also those expressed in the sudden infant death syndrome susceptibility pathway. This may help clarify the relationship between sudden death with intratumoral hemorrhage in undiagnosed subclinical IHG.

The prognosis in IHG is generally better than in other pediatric high-grade gliomas. The majority of described patients have been treated with surgical resection and chemotherapy. However, surgically related adverse events are frequent in children with IHG^1,5^; in our cohort, 2 patients died of intraoperative hemorrhage at the tumor bed after radical resection of the tumor, and 1 patient died of massive bleeding during a repeated surgical undertaken after disease progression. Potentially, early detection of the tumor by neurosonography, which increases the likelihood of successful radical resection of the tumor, may result in better outcomes, although this needs to be proven.

Cytotoxic chemotherapy for IHG is usually of limited efficacy, as the majority of patients develop progression of the disease during or after completion of the chemotherapy.^1,6^ Only 2 of the patients in this series are currently in long-term remission after gross total resection and completion of the chemotherapy. Of note, 2 patients, not given chemotherapy after the initial diagnosis of DIA (subsequently altered to IHG based on molecular findings), experienced a long-term remission of the disease after radical surgery only. A case of IHG in a 3-year-old girl, with similar histology and harboring a similar transcript has been published; the child attained durable remission with resection alone.^8^ Despite the higher survival rates with radical tumor resection, the risk of developing immediate life-threatening or late complications makes a radical resection impossible in some situations and underscores the potential benefit of targeted therapy in the neoadjuvant setting.

Overall, molecularly targeted therapy with tyrosine-kinase inhibitors appears effective in IHG. The incidence of therapeutic targets for small-molecule tyrosine-kinase inhibitors in IHG is estimated to be 72%-83%,^6,26^ which is consistent with the 100% incidence in our cohort (*n* = 15). In the STARTRK-NG trial, entrectinib afforded pronounced clinical response in the majority of patients with *NTRK1/2/3-, ROS1-* or *ALK*-rearranged solid tumors.^7^ The efficacy of tyrosine kinase inhibitors (entrectinib, larotrectinib, alectinib) specifically in IHG has been demonstrated in a number of published observations.^6–12,20,21^ In most reports on targeted therapy in IHG, the observation period was limited to several months after achieving the clinical effect. The experience with lorlatinib in IHG is confined to isolated clinical cases.^13–16^ In the current study, lorlatinib was used as a next-generation ROS1 inhibitor in a patient developing progression on entrectinib, as well as in single cases after disease progression on chemotherapy or as first-line therapy.

## Conclusion

Infant-type hemispheric gliomas (IHG) are rare glial tumors that develop in utero or possibly infancy. Standard treatments for IHG have limited efficacy. Due to the large size of the tumor masses and their high vascularity surgery may be subtotal. Similarly results of therapy with cytostatic drugs is variable and often suboptimal. Molecular-genetic methods allow unambiguous verification of the diagnosis in IHG. Moreover, characteristic fusion transcripts identifiable by RNA sequencing provide a rationale for molecularly targeted therapy, which currently appears the most efficacious modality of treatment for patients with IHG.

## Data Availability

The data supporting findings could be provided from corresponding author upon reasonable request.
